# Spinal TNF-α receptor 1 is differentially required for phrenic long-term facilitation (pLTF) over the course of motor neuron death in adult rats

**DOI:** 10.3389/fphys.2024.1488951

**Published:** 2024-12-05

**Authors:** Ryan D. Lewis, Amy N. Keilholz, Catherine L. Smith, Ethan A. Burd, Nicole L. Nichols

**Affiliations:** ^1^ Department of Biological Chemistry, Grinnell College, Grinnell, IA, United States; ^2^ Department of Veterinary Pathobiology, College of Veterinary Medicine, University of Missouri, Columbia, MO, United States; ^3^ Department of Biomedical Sciences, College of Veterinary Medicine, University of Missouri, Columbia, MO, United States; ^4^ Department of Biology, Seton Hill University, Greensburg, PA, United States; ^5^ Department of Medical Pharmacology and Physiology, School of Medicine, University of Missouri, Columbia, MO, United States; ^6^ Dalton Cardiovascular Research Center, University of Missouri, Columbia, MO, United States

**Keywords:** phrenic motor neuron death, breathing, respiration, plasticity, astrocyte, rat model

## Abstract

**Introduction:**

Intrapleural injections of cholera toxin B conjugated to saporin (CTB-SAP) result in selective respiratory (*e.g.*, phrenic) motor neuron death and mimics aspects of motor neuron disease [(*e.g.*, amyotrophic lateral sclerosis (ALS) and spinal muscular atrophy (SMA)], such as breathing deficits. This rodent model allows us to study the impact motor neuron death has on the output of surviving phrenic motor neurons as well as the compensatory mechanisms that are recruited. Microglial density in the phrenic motor nucleus as well as cervical gene expression of markers associated with inflammation (*e.g*., tumor necrosis factor α; TNF-α) are increased following CTB-SAP-induced phrenic motor neuron death, and ketoprofen (nonsteroidal anti-inflammatory drug) delivery attenuated phrenic long-term facilitation (pLTF) in 7 day (d) CTB-SAP rats but enhanced pLTF in 28d CTB-SAP rats.

**Methods:**

Here, we worked to determine the impact of TNF-α in the phrenic motor nucleus by: 1) quantifying TNFR1 (a high affinity transmembrane receptor for TNF-α) expression; 2) investigating astrocytes (glial cells known to release TNF-α) by performing a morphological analysis in the phrenic motor nucleus; and 3) determining whether acute TNFR1 inhibition differentially affects phrenic plasticity over the course of CTB-SAP-induced motor neuron loss by delivering an inhibitor for TNF-α receptor 1 (sTNFR1i) in 7d and 28d male CTB-SAP and control rats.

**Results:**

Results revealed that TNFR1 expression was increased on phrenic motor neurons of 28d CTB-SAP rats (*p* < 0.05), and that astrocytes were increased and exhibited reactive morphology (consistent with an activated phenotype; *p* < 0.05) in the phrenic motor nucleus of CTB-SAP rats. Additionally, we found that pLTF was attenuated in 7d CTB-SAP rats but enhanced in 28d CTB-SAP rats (*p* < 0.05) following intrathecal sTNFR1i delivery.

**Conclusion:**

This work suggests that we could harness TNFR1 as a potential therapeutic agent in CTB-SAP rats and patients with respiratory motor neuron disease by increasing compensatory plasticity in surviving neurons to improve phrenic motor neuron function and breathing as well as quality of life. Future studies will focus on microglial and astrocytic cytokine release, the role they play in the differential mechanisms of pLTF utilized by 7d and 28d CTB-SAP rats, and potential therapies that target them.

## Introduction

Neuromuscular/neurodegenerative diseases such as spinal muscular atrophy (SMA), amyotrophic lateral sclerosis (ALS), and some spinal cord injuries manifest as respiratory motor neuron loss, diminished respiratory motor output, and ventilatory failure resulting in death. (Bourke et al., 2001; Lyall et al., 2001; Lechtzin et al., 2002; Nogues et al., 2002; Wong et al., 2002; Nichols et al., 2013; Boentert et al., 2017). The respiratory system is capable of remarkable plasticity despite respiratory motor neuron loss, but eventually these mechanisms fail. Mechanisms underlying this plasticity remain poorly understood, and current animal models do not allow study of only respiratory motor neuron loss. Thus, to study the effects caused by respiratory motor neuron loss, we utilized our established model of bilateral, intrapleural injections of cholera toxin B conjugated to saporin (CTB-SAP) to create an inducible rodent model of targeted and controlled respiratory (*e.g.*, phrenic) motor neuron death (Nichol et al., 2015; Nichols et al., 2018; Borkowski and Nichols, 2020). In response to respiratory motor neuron loss, eupneic breathing is preserved but deficits in maximal ventilatory capacity are observed in this model (Nichols et al., 2015; Nichols et al., 2018). This suggests that mechanisms of neuroplasticity may help maintain eupneic ventilation during respiratory motor neuron loss, and that harnessing these mechanisms as a therapeutic strategy may preserve maximal ventilatory capacity.

Intrapleurally injected CTB-SAP results in phrenic motor neuron loss and increased microglial density in the phrenic motor nucleus (Nichols et al., 2015; Nichols et al., 2018). We speculate that surviving respiratory motor neurons utilize mechanisms of plasticity, such as phrenic motor facilitation (pMF), to maintain eupnea (Mitchell et al., 2001). Phrenic long-term facilitation (pLTF) is a form of pMF induced by acute intermittent hypoxia (AIH). In control rats, moderate AIH (*i.e.*, 35–55 mmHg PaO2) elicits pLTF through Gq-dependent mechanisms via serotonin two receptors (5-HT2) in the phrenic motor nucleus (Bach and Mitchell, 1996; Baker-Herman et al., 2004; Hoffman et al., 2012; Devinney et al., 2013; Devinney et al., 2015), while severe AIH (*i.e.*, 25–35 mmHg PaO2) elicits enhanced pLTF through a different pathway using Gs-dependent mechanisms and adenosine (A2A) receptors (Golder et al., 2008; Nichols et al., 2012; Devinney et al., 2013; Agosto-Marlin and Mitchell, 2017). Interestingly, 7 day (d) CTB-SAP rats benefit from enhanced pLTF from the Gs-pathway, while 28d CTB-SAP rats utilize the Gq-pathway for moderate pLTF (Nichols et al., 2013; Borkowski and Nichols, 2020; Borkowski et al., 2021).

Microglial activation and inflammation are hallmarks of neurodegenerative disorders, but it remains unknown whether this activation is beneficial or detrimental over the course of neuropathology (Beers et al., 2006; Boillee et al., 2006; Beers et al., 2011; Philips and Robberecht, 2011). To investigate why the mechanisms and magnitude of plasticity are differentially affected over time, we previously utilized ketoprofen (a non-steroidal anti-inflammatory drug; NSAID) to explore the role of cyclooxygenase-1/2 (COX-1/2)-induced inflammation in respiratory plasticity. The results of this study showed that COX-1/2-induced inflammation differentially contributes to respiratory plasticity and may play a role in CTB-SAP rats to maintain phrenic plasticity in the face of acute phrenic motor neuron loss at day 7 or constrain respiratory plasticity in the presence of chronic phrenic motor neuron loss at day 28 (Borkowski et al., 2022). Additionally, we conducted a cervical spinal inflammatory-associated marker study that surveyed a set of signaling molecules that could be involved. The only marker tested that was significantly different at both time points was Tumor Necrosis Factor alpha (TNF-α) (Borkowski et al., 2022). TNF-α is often released from glial cells, such as astrocytes and microglia, when reacting to motor neuron damage or death. When the CNS sustains damage, microglia will proliferate, migrate to the injury site, transition from ramified to active (amoeboid) morphology (Kreutzberg, 1996; Graeber, 2010), and release inflammatory-associated molecules like TNF-α (Huston and Tracey, 2011). These studies demonstrated that microglial number is increased, and an amoeboid morphology (*i.e.*, retracted processes, enlarged cell body) consistent with an activated phenotype is exhibited in the phrenic motor nucleus of 7d and 28d CTB-SAP rats suggesting that microglia could be a source of increased TNF-α production (Borkowski et al., 2022).

The goals of the current study were to further determine the potential impact of TNF-α in the phrenic motor nucleus and explore whether acute delivery of an inhibitor for the high affinity TNF-α transmembrane receptor, tumor necrosis factor receptor 1 (TNFR1), via recombinant human soluble TNF receptor 1 (sTNFR1i) differentially affects respiratory (*i.e.*, phrenic) plasticity over the course of CTB-SAP-induced motor neuron loss. Here, we quantified the expression of TNFR1, inside the phrenic motor nucleus and the non-phrenic motor nucleus (area immediately surrounding the phrenic motor nucleus), specifically on phrenic motor neurons and glial cells (astrocytes and microglia) in 7d and 28d CTB-SAP rats vs. controls. Furthermore, we conducted a morphological analysis of astrocytes in the phrenic motor nucleus and non-phrenic motor nucleus. Finally, sTNFR1i was used to investigate the effects of sTNFR1i on pLTF in 7d and 28d CTB-SAP rats vs. controls. We hypothesized that: 1) TNFR1 expression will be increased in the phrenic motor nucleus, specifically on phrenic motor neurons in CTB-SAP rats vs. controls; 2) astrocyte number will be increased in the phrenic motor nucleus, and astrocyte morphology will be consistent with a reactive morphology in CTB-SAP rats vs. controls; and 3) enhanced pLTF in 7d CTB-SAP rats will be attenuated when pre-treated with sTNFR1i, while pLTF will be enhanced in 28d CTB-SAP rats pre-treated with sTNFR1i. This research may provide insight into how mechanisms of plasticity are altered over the course of disease progression. Additionally, by understanding the differential mechanisms involved and the impact inflammation has on respiratory plasticity, we can more accurately treat patients by modulating neuroinflammation throughout respiratory motor neuron disease progression to maximize breathing.

## Materials and methods

### Animals

Experiments were conducted on adult (3–4 months old) male Sprague Dawley rats (Envigo Colony 208a; Indianapolis, IN, United States). The rats were pair-housed in standard vivarium conditions (*i.e.*, ambient temperature 20°–26°C, humidity 30%–70%, and standard 12:12 light:dark cycle) and had access to a standard commercial pelleted diet and water *ad libitum*. Animal care staff performed daily health monitoring and routine surveillance for common rodent ailments. All experimental procedures were approved by the University of Missouri’s Institutional Animal Care and Use Committee (IACUC) and conducted in accordance with the Guide for the Care and Use of Laboratory Animals within our USDA-licensed and AAALAC-accredited academic institution.

### Experimental procedures

Rats were randomly allocated to four experimental groups to either study the effects of cholera toxin B conjugated to saporin (CTB-SAP; Advanced Targeting Systems) on phrenic tumor necrosis factor receptor 1 (TNFR1) expression and astrocyte morphology (n = 31) or study pLTF following sTNFR1i or vehicle delivery (n = 94). All rats received bilateral, intrapleural injections of either 25 μg unconjugated CTB + SAP (*i.e.*, control) or conjugated CTB-SAP, studied 7 or 28 days later, and then subsequently euthanized using American Veterinary Medical Association (AVMA) approved methods. Specifically, rats were euthanized via exsanguination under deep isoflurane anesthesia by transcardial perfusion with cold PBS/saline (0.9% NaCl in 0.01 M sodium phosphate buffer, pH 7.0) followed by paraformaldehyde (in 0.1 M sodium phosphate buffer, pH 7.4) for collection of tissue and histological analysis. In all cases, death was confirmed via bilateral pneumothorax where the chest cavity was opened, and lack of respiration and heartbeat were confirmed.

#### Intrapleural injections to create an inducible rat model of selective respiratory motor neuron death

Our rat model was created as previously described (Borkowski et al., 2022). In brief, Rats received bilateral intrapleural injections (6 mm deep, fifth intercostal space) using a 50 μL Hamilton syringe and a custom needle (6 mm, 23 gauge, semi-blunt to prevent lung puncture) while under isoflurane anesthesia (1.5% in 100% oxygen as previously described) (Mantilla et al., 2009). Control rats received bilateral intrapleural injections with cholera toxin B subunit (CTB to retrogradely label phrenic motor neurons; 20 μg dissolved in ddH2O; Calbiochem; Billerica, MA) unconjugated to saporin (SAP; 25 μg dissolved in phosphate buffered saline (PBS); Advanced Targeting Systems; San Diego, CA) to facilitate comparisons when examining respiratory plasticity, astrocyte morphology, and cervical spinal TNFR1 expression. CTB conjugated to saporin (CTB-SAP; 25 μg dissolved in PBS; Advanced Targeting Systems; San Diego, CA) was intrapleurally injected to target respiratory motor neurons as described previously (Nichols et al., 2015; Nichols et al., 2018; Borkowski and Nichols, 2020). CTB-SAP rats also received an additional 20 μg of CTB dissolved in ddH2O (Calbiochem; Billerica, MA) in their administered intrapleural injections to retrogradely label surviving phrenic motor neurons. Rats were closely monitored for any signs of respiratory distress (*i.e*., shortness of breath, labored breathing) following intrapleural injections and housed for 7 or 28 days before either tissue was collected and prepared for immunohistochemistry or surgical preparation and *in vivo* neurophysiological experiments were performed as described below.

#### Immunohistochemistry

Tissue used for staining was obtained from a separate set of rats than those used for the *in vivo* neurophysiological studies and did not receive any drug or AIH treatment during neurophysiological experiments. Rats were transcardially perfused and spinal cord sections were post-fixed, transversely sectioned and stored at −20°C as previously described (Borkowski et al., 2021; Borkowski et al., 2022). Six sections (40 μm thick) from the cervical spinal cord (C4) were selected for each animal (4 groups; n = 8 per group) to evaluate TNFR1 expression and astrocytic morphology in the phrenic motor nucleus of 7 day (d) and 28d control and CTB-SAP rats. The tissue was washed with 1× PBS three times for 5 minutes on a shaker at room temperature (RT). An antigen retrieval protocol was performed to increase the robustness of TNFR1 using a 0.01 M citrate in ddH2O added to the tissue wells and put on the shaker for 30 min at 60°C. The tissue was washed again in 1X PBS 3 × 5 min on shaker at RT before sections were then incubated at RT in a blocker solution (1× PBS +0.2% Triton +5% normal donkey serum) at RT on a shaker for 1 h. Sections were then incubated in primary antibody solution [1X PBS +0.1% Triton +5% normal donkey serum + antibodies against cholera toxin B (CTB; goat polyclonal, 1:2k, Sigma, catalog #227040), TNFR1 (rabbit polyclonal, 1:250, Abcam, catalog #AB223352), CD11b (mouse monoclonal, 1:500, BioRad, catalog #MCA2759; to detect microglia), or GFAP (mouse monoclonal, 1:800, Sigma, catalog #G3893; to detect astrocytes)] overnight at 4°C on a shaker. The next day, tissue was washed three times for 5 min at RT on a shaker in 1× PBS. The tissue was then incubated in secondary antibody solution (1X PBS +0.1% Triton +5% normal donkey serum + donkey anti-goat Alexa-Fluor 555 (1:1k; Molecular Probes, catalog #A21432), donkey anti-rabbit Alexa-Fluor 488 (1:1k; Molecular Probes, catalog #A21206)), and donkey anti-mouse Alexa-Fluor 647 (1:1k; Molecular Probes, catalog #A31571) for 2 h on a shaker in the dark at RT. The tissue was washed again while covered in 1× PBS three times for 5 min on a shaker at RT before being mounted on positively charged glass slides (Thermo Fisher Scientific, Waltham, MA) and allowed to dry before ProLong™ Gold anti-fade reagent (Thermo Fisher Scientific, Waltham, MA) was applied, and a coverslip was put onto the slides. Covered slides were stored at 4°C until quantification of staining was performed.

#### Imaging

Photomicrographs were taken at the same settings for all images per antibody per group [*i.e.,* gains for images: 555 (CTB) = 812.2; 488 (TNFR1) = 798.7; 647 (Cd11b) = 841.5; 647 (GFAP) = 841.5]. The photomicrographs were taken using a Leica DM4000 confocal microscope at × 20 magnification with Leica Application Suite X (LAS X) software to encompass the CTB-labelled phrenic motor neurons in the C4 cervical spinal cord (Boulenguez et al., 2007; Mantilla et al., 2009; Watson et al., 2009; Nichols et al., 2015). Images were taken as 8-bit stacks at a resolution of 1024 × 1024 (366.67 μm × 366.67 μm).

#### TNFR1 expression and astrocytic morphological analysis using ImageJ and IMARIS

ImageJ software was used to create Z stacks and quantify TNFR1 expression on CTB stained phrenic motor neurons, microglia, and astrocytes inside and outside the phrenic motor nucleus (PMN) for all experimental groups (n = 31) as follows: 1) 7d control (n = 7); 2) 7d CTB-SAP (n = 8); 28d control (n = 8); and 28d CTB-SAP (n = 8) rats. Z stacks of all photomicrographs from rats stained for astrocytes were then opened in IMARIS software for astrocytic morphological analysis for the following groups: 1) 7d control (n = 3); 2) 7d CTB-SAP (n = 4); 28d control (n = 4); and 28d CTB-SAP (n = 4) rats. The region of interest (ROI) set for the phrenic motor nucleus was used to create a ROI surface through the stack to quantify astrocyte morphology inside and outside the phrenic motor nucleus. Once the high-resolution 3D surface was created, values outside the ROI were masked to isolate the GFAP + regions of interest. Next, starting points were placed in the cell body of each astrocyte within the ROI, and seed points were placed along the projections allowing the software to automatically create a 3D reconstruction of each astrocyte in the ROI to enable a comprehensive morphological analysis. Filament length sum (primary projections from cell body), branch length (secondary projections), branch volume sum, filament volume sum, number of branches, number of end-points, filament distance from the origin sum, and the number of astrocytes within the ROI were quantified as previously described (Cengizr et al., 2019; Borkowski et al., 2022). Additionally, a Sholl analysis was performed to calculate branching density within the ROI by determining the number of intersections the branches make and at what distance these intersections occur from the origin of the cell body using a series of concentric circles (1 μm apart). (From the Sholl analysis, the process maximum (Nm, the maximum number of intersections for the cell), and the critical value (Cr, the distance from the cell body where Nm occurred) per astrocyte were evaluated as previously described (Morrison and Filosa, 2013; Borkowski et al., 2022). An algorithm written in Visual Studio Code was used to average all parameters across astrocytes within each ROI, per animal and subsequently per group.

#### Surgical preparation for *in vivo* neurophysiological experiments

Experimental procedures for *in vivo* neurophysiology were performed as described previously (Hoffman et al., 2012; Nichols et al., 2018). In short, rats were isoflurane anesthetized (3.5% in 50% O_2_, balance N_2_), tracheotomized, and pump ventilated (Small Animal SAR-1000 Ventilator; CWE, Ardmore, PA, USA; tidal volume ∼2.5 mL, frequency ∼70–80 breaths per minute) throughout surgical procedures prior to being converted to urethane (1.85 g kg− 1, i. v.) anesthesia over 15–20 min while isoflurane was gradually withdrawn. Next, rats were paralyzed *via* muscular blockade using pancuronium bromide (2.5 mg kg− 1, i. v.). To maintain bodily fluid concentrations and electrolyte balance, rats were given a 1:2:0.13 mixture of 6% Hetastarch (in 0.9% sodium chloride), lactated Ringer’s solution, and 8.4% sodium bicarbonate via continuous intravenous infusion (1.5–6 mL/kg/hour). Anesthetic depth was confirmed via toe pinch using a hemostat prior to beginning and immediately after surgical and neurophysiological protocols were complete. Body temperature was maintained (37◦C ± 1°C) with a custom-made heated surgical table, and was assessed with a rectal thermometer (Physitemp, Clifton, NJ, USA). Throughout the surgical preparation, end-tidal PCO_2_ (PETCO_2_) was maintained at ∼45 mmHg and monitored with a flowthrough carbon dioxide analyzer designed to sufficiently measure response time for PETCO_2_ measurements in rats (CapStar-100, CWE, Ardmore, PA). Rats were bilaterally vagotomized and blood pressure was monitored in the right femoral artery by the insertion of a polyethylene catheter (PE50 ID: 0.58 mm, OD: 0.965 mm; Intramedic, MD, USA) connected to a pressure transducer (APT300 Pressure Transducer, Harvard Apparatus, Holliston, MA, USA). Arterial blood samples were taken during baseline, the first hypoxic episode, and at 15, 30 and at 60 min post-AIH and analyzed for partial pressures of O_2_ (PO_2_) and CO_2_ (PCO_2_) using a blood gas analyzer (ABL80 Flex, Radiometer, Brea, CA). Through a dorsal approach, the left phrenic nerve was isolated, distally cut, desheathed, and covered with a saline soaked cotton ball until it was placed on a bipolar silver electrode to record nerve activity. For intrathecal drug delivery, a dorsomedial incision was first made along the cervical spinal column and the muscle was removed over the C2–3 vertebrate, and a subsequent laminectomy was performed at C2. A small incision was then made in the dura, and a soft silicone catheter (2 Fr; Access Technologies, Skokie, IL) was inserted subdurally 3–4 mm caudal from the incision until the tip rested over the C4 segment. Vehicle or sTNFR1i (see below) were then delivered from a 50 μL Hamilton syringe. Due to the dampening effects that isoflurane has on phrenic nerve output, a minimum of 1 h was permitted after the conversion to urethane anesthesia before *in vivo* neurophysiological recordings began to eliminate this effect.

#### *In Vivo* neurophysiological recordings

Nerve activity was recorded as described previously (Hoffman et al., 2012; Nichols et al., 2018) where the left phrenic nerve was submerged in mineral oil and placed on bipolar silver electrodes to record nerve activity, and then neural signals were amplified band-pass filtered, full-wave rectified, integrated, digitized and analyzed using a WINDAQ data acquisition system (DATAQ Instruments, Akron, OH, USA). Apneic and recruitment thresholds as well as baseline conditions were determined as previously reported (Hoffman et al., 2012; Nichols et al., 2018) Following baseline measurements, rats were exposed to three 5-min episodes of isocapnic (±1.5 mmHg) acute intermittent hypoxia [10% inspired O_2_, arterial PO_2_ (PaO_2_): 35–45 mmHg] separated by 5-min intervals of baseline O_2_ conditions (50% inspired O_2_, PaO_2_: ≥150 mmHg) or were time controls (TC; no AIH). Rats were returned to baseline inspired O_2_ levels after the third bout of hypoxia, and inspired O_2_ levels were maintained for the duration of the experiments. Phrenic nerve output was measured for approximately 60 min following the AIH protocol, and inspired CO_2_ and/or the ventilation rate were manipulated to maintain isocapnic arterial PCO2 (PaCO2) within ±1.5 mmHg of the respective baseline value.

In order to test the hypothesis that TNF-α signaling plays a role in the differential pLTF observed in 7d and 28d CTB-SAP rats, rats received an acute intrathecal delivery (3–4 μL boluses over 2 min) of vehicle (artificial cerebral spinal fluid; aCSF) or 0.13 μg/μL an inhibitor for TNFR1 (recombinant human soluble TNF receptor 1; R&D Systems, Minneapolis, MN, USA) (Broytman et al., 2013; Baertsch and Baker-Herman, 2015) 20 min prior to the acute intermittent hypoxia (AIH) protocol. Eight control and CTB-SAP groups were tested and experimental group assignments are summarized in Table 1. Since multiple previous studies have demonstrated slight experimental drift in TC groups (no AIH), and there were no apparent differences in TC groups in this study, all TC treated rats were grouped together by treatment (*i.e.*, sTNFR1i and veh) within control and CTB-SAP rats for statistical analyses. This was done to ensure that sTNFR1i and the vehicle did not elicit unexpected pMF (*i.e.*, a time-dependent drift in time control experiments), while minimizing animal use consistent with national standards for animal research.

**TABLE 1:** *In Vivo* Neurophysiological Experimental Group Assignments [vehicle (veh); inhibitor for tumor necrosis factor alpha receptor 1 (recombinant human soluble TNFR1; sTNFR1i); without AIH (time-control or TC)].

Experimental groups		Sample size
Control	7d Veh	8
	7d sTNFR1i	8
	28d Veh	9
	28d sTNFR1i	9
	7d Veh TC	4
	7d sTNFR1i TC	5
	28d Veh TC	4
	28d sTNFR1i TC	5
CTB-SAP	7d Veh	7
	7d sTNFR1i	8
	28d Veh	6
	28d sTNFR1i	8
	7d Veh TC	3
	7d sTNFR1i TC	3
	28d Veh TC	4
	28d sTNFR1i TC	3
Total		94

#### Statistical analysis

Investigators involved in data collection were blinded to experimental group assignment. For TNFR1 expression analysis, TNFR1 (+) pixels were quantified on CTB (+) phrenic motor neurons as well as glial cells and statistically compared within and between control and CTB-SAP groups using a one-way ANOVA. TNFR1 expression was not different between 7d and 28d controls, so they were combined together. For astrocytic morphology, a one-way ANOVA was used to compare 7d and 28d timepoints which were not statistically different for both CTB-SAP rats and controls for all parameters (*p* > 0.05), so they were combined for each group and compared using a Student’s t-test to compare CTB-SAP rats vs. controls for each parameter. For the *in vivo* neurophysiology studies, the change in phrenic amplitude (percent baseline), was not different within time control groups so they were combined before conducting a two-way ANOVA with repeated measures test for pLTF and blood gases. A one-way ANOVA was also used to compare hypoxia responses as well as percent baseline comparisons at 60 min post-hypoxia. When significant differences were detected, a Fisher’s LSD *post hoc* analysis was conducted. Differences between groups were considered significant if *p* < 0.05, and all values were expressed as means ± 1 SEM.

## Results

### TNFR1 expression is increased on phrenic motor neurons in 28d CTB-SAP rats

**FIGURE 1:**
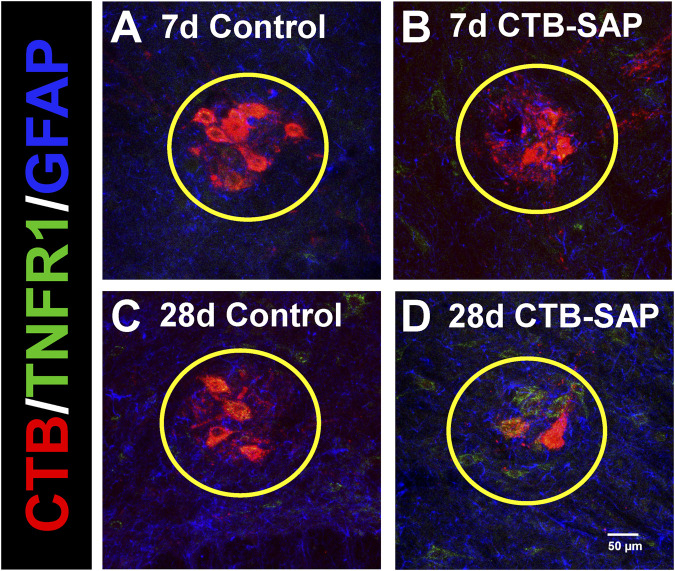
Representative photomicrographs displaying TNFR1 expression within the phrenic motor nucleus and non-phrenic motor nucleus in 7d and 28d control **(A, C)** and CTB-SAP **(B, D)** rats from C4 spinal cord sections. The representative images highlight phrenic motor neurons (CTB; red), TNFR1 expression (green), and astrocyte expression (GFAP; blue) inside the phrenic motor nucleus (area within yellow circle) and the non-phrenic motor nucleus (area outside of the yellow circle) from C4 spinal cord sections in 7d and 28d control **(A, C)** and CTB-SAP **(B, D)** rats at ×20 magnification.

**FIGURE 2:**
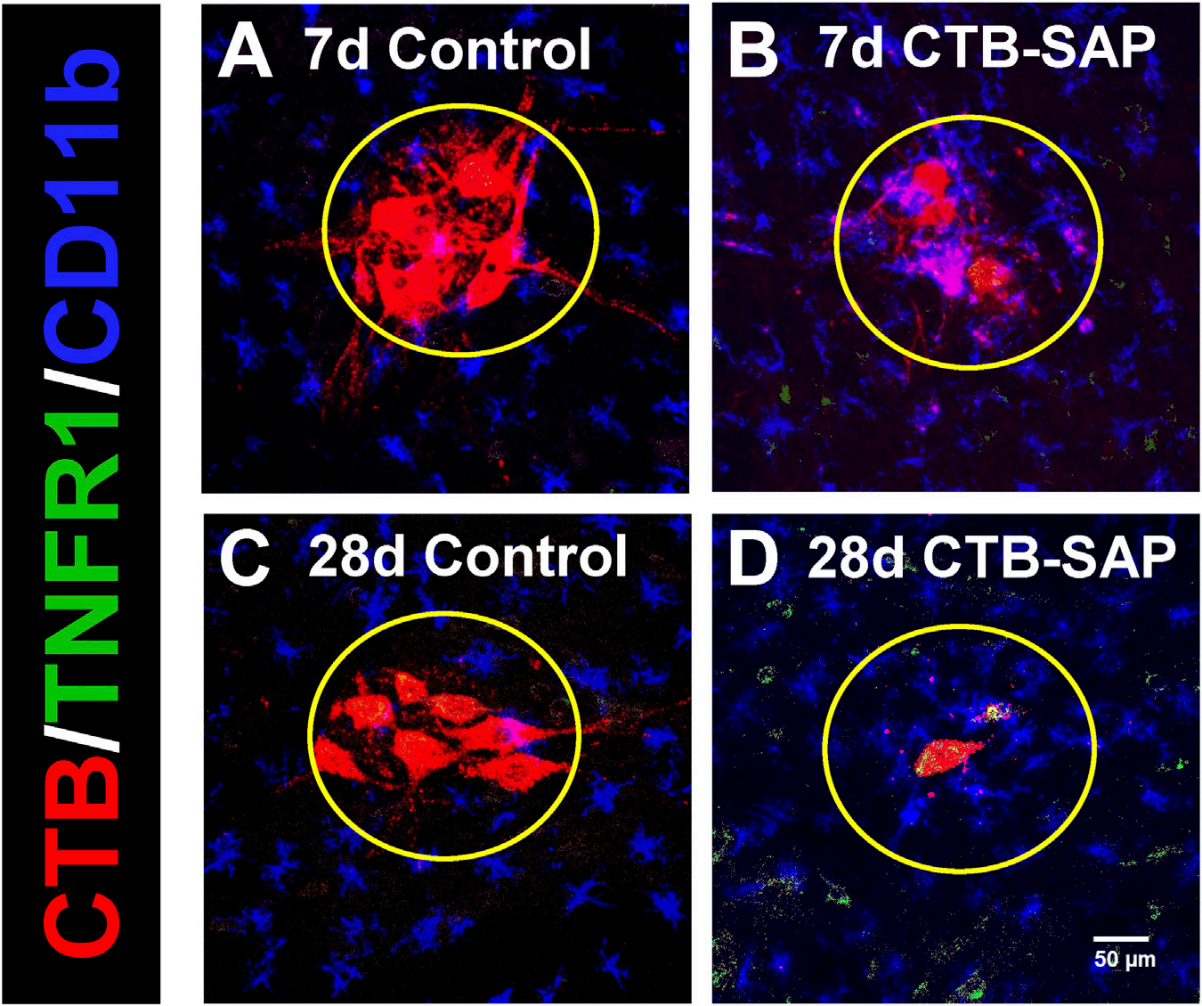
Representative photomicrographs displaying TNFR1 expression within the phrenic motor nucleus and non-phrenic motor nucleus in 7d and 28d control **(A, C)** and CTB-SAP **(B, D)** rats from C4 spinal cord sections. The representative images highlight phrenic motor neurons (CTB; red), TNFR1 expression (green), and microglial expression (CD11b; blue) inside the phrenic motor nucleus (area within yellow circle) and the non-phrenic motor nucleus (area outside of the yellow circle) from C4 spinal cord sections in 7d and 28d control **(A, C)** and CTB-SAP **(B, D)** rats at ×20 magnification.

**FIGURE 3:**
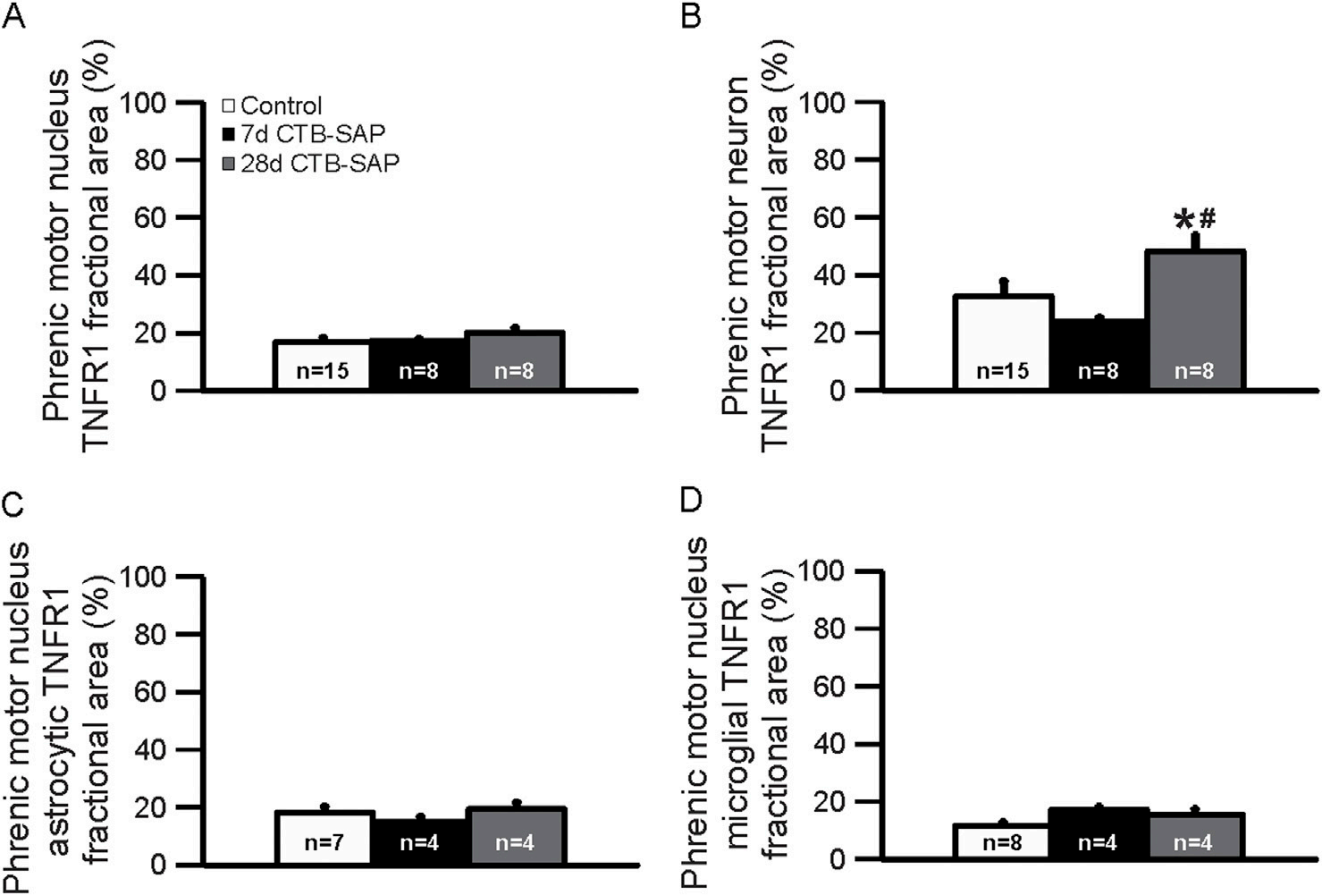
Immunohistochemical quantification (fractional area) of TNFR1 expression in the phrenic motor nucleus **(A)** and on phrenic motor neurons **(B)**, astrocytes **(C)**, and microglia **(D)** in 7d and 28d control and CTB-SAP rats in C4 spinal cord sections. TNFR1 expression was not different between 7d and 28d controls, so they were combined together. TNFR1 expression is increased on phrenic motor neurons in 28d CTB-SAP rats (gray) vs. controls (white; *) and 7d CTB-SAP rats (black; #) but is otherwise unaffected across groups in the phrenic motor nucleus and non-phrenic motor nucleus **(A–D)**. Values are expressed as means ±1 S.E.M, and differences were considered significant if *p* < 0.05.

TNFR1 expression was measured in 7d and 28d CTB-SAP rats vs. controls in the phrenic motor nucleus and non-phrenic motor nucleus (*i.e.*, area immediately outside the phrenic motor nucleus), specifically on phrenic motor neurons as well as glial cells (astrocytes and microglia). TNFR1 expression was significantly increased on phrenic motor neurons in 28d CTB-SAP rats vs. controls and 7d CTB-SAP rats (Figures 1–3; Supplemental Figures S1, 2; *p* < 0.05). No significant differences were found across timepoints inside or outside the phrenic motor nucleus for TNFR1 expression on astrocytes or microglia (Figures 1–3; Supplemental Figures S1, 2; *p* > 0.05).

### Astrocytic morphological changes are exhibited in CTB-SAP rats vs. controls

**FIGURE 4:**
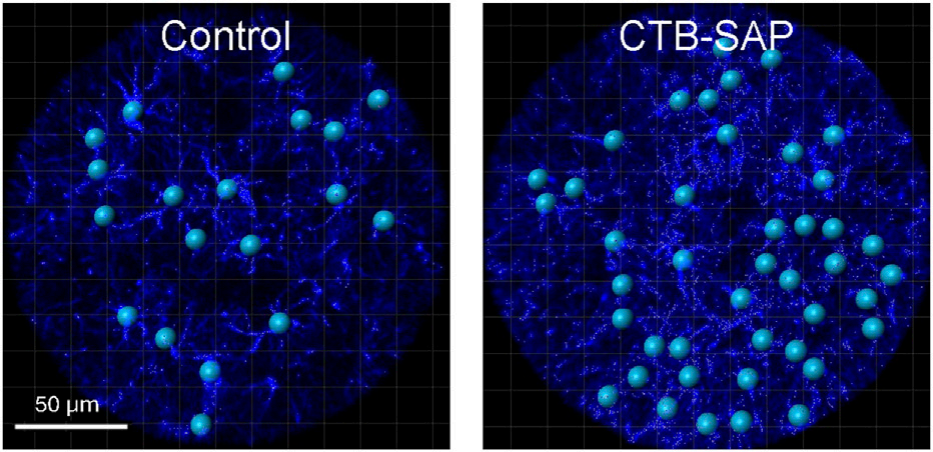
Representative images of the morphological analysis of astrocytes (GFAP; blue) in the phrenic motor nucleus from C4 cervical spinal cord segments from a control and CTB-SAP rat using IMARIS software. Images taken of C4 cervical spinal cord segments were used to make z-stacks and rendered into 3D models of GFAP (blue) labelled astrocytes. The surface feature was used to generate the phrenic motor nucleus region of interest which was used to specifically separate values outside and inside the phrenic motor nucleus through a masking process. The filament feature was used to render the 3D models by placing starting points (blue spheres) at the center of each astrocyte and seed points (small white spheres) along astrocyte projections that were used to trace each filament. Following filament creation, astrocyte models were quantified inside and outside the phrenic motor nucleus. The resulting data was processed for filament and branch length and volume (*e.g.,* the closer the seed points are to the starting point, the shorter the projections are from the cell body of the astrocyte, which demonstrates a reactive morphological state), and a Sholl analysis was performed using an algorithm written in Visual Studio Code to determine the number of intersections and intersection distance (see Table 2).

IMARIS software was used to evaluate morphological changes in astrocytes within the phrenic motor nucleus of CTB-SAP rats vs. controls. 7d and 28d timepoints were not statistically different for both CTB-SAP rats and controls (*p* > 0.05), so they were combined for each group. Increased astrocyte number as well as parameters indicative of a reactive morphological state (*e.g.*, increased branch number, decreased branch length) were observed in CTB-SAP rats vs. controls. Specifically, astrocyte number is significantly increased (*p* < 0.05) and astrocytes exhibited an altered morphology (*e.g.,* significantly increased branch number, number of end points, filament volume, and intersection number, as well as significantly decreased branch volume and branch length; Figure 4; Table 2; *p* < 0.05) consistent with a reactive phenotype in the phrenic motor nucleus of CTB-SAP rats vs. controls. Additionally, astrocyte number was significantly increased (*p* < 0.05) and branch length was significantly decreased in the non-phrenic motor nucleus (Figure 4; Table 2).

**TABLE 2:** Astrocytic morphological analysis inside and outside the phrenic motor nucleus (PMN) of CTB-SAP rats vs. controls. 7d and 28d timepoints were not statistically different for both CTB-SAP rats and controls (*p* > 0.05), so they were combined for each group. Astrocyte number, filament volume, branch number, number of intersections, and end-points are increased, whereas branch volume and length are decreased in the PMN of CTB-SAP rats vs. controls. These results are consistent with a reactive (activated) morphology. Values are expressed as means ±1 S.E.M, and differences were considered significant if *p* < 0.05.

Experimental groups	Average branch length (µm)	Average branch number	Average branch volume (µm^3^)	Number of end-points	Number of astrocytes	Average filament length sum (µm)	Average filament volume sum (µm^3^)	Number of intersections	Intersection distance (µm)	Filament distance from origin (µm)
Inside PMN										
Control	19 ± 3	3 ± 0.3	197 ± 12	5 ± 0.4	25 ± 3	129 ± 13	1294 ± 66	4 ± 0.2	25 ± 3	272 ± 4
CTB-SAP	12 ± 0.4*	4 ± 0.4*	154 ± 8*	7 ± 0.5*	36 ± 1*	132 ± 8	1570 ± 44*	6 ± 0.4*	23 ± 0.3	265 ± 3
Outside PMN										
Control	20 ± 3	2 ± 0.2	205 ± 9	4 ± 0.3	63 ± 10	114 ± 19	1063 ± 35	3 ± 0.1	25 ± 3	302 ± 3
CTB-SAP	15 ± 1*	2 ± 0.3	189 ± 11	5 ± 0.4	92 ± 7*	99 ± 11	1182 ± 52	4 ± 0.2	19 ± 1	301 ± 5

### Blood gases and mean arterial pressures

Arterial PCO_2_ (PaCO_2_) was successfully maintained within 1.5 mmHg from its baseline value in all groups (Table 3) despite small and significant changes within and across groups, therefore the changes we observed in pLTF are not attributed to differences in chemoreceptor feedback (*i.e.,* changes >1.5 mmHg of baseline in PaCO_2_ can influence pLTF; Bach and Mitchell, 1996). During AIH, arterial PaO_2_ (PaO_2_) was maintained successfully within the target range (35–45 mmHg) and remained above 150 mmHg at all time points following hypoxia (Table 3). In addition, mean arterial pressure differed among groups when AIH vs. TCs were compared during hypoxic episodes as expected, and slight but significant differences within groups were ∼20 mmHg or less at 60 min post-hypoxia, and this was consistent among groups Table 3; changes in mean arterial pressure of ∼20 mmHg or less from baseline values have minimal effect on respiratory activity in rats (Bach and Mitchell, 1996; Walker and Jennings, 1996)]. Thus, the pLTF expression differences that we observed were not affected by changes in PaCO_2_, PaO_2_, or blood pressure regulation.

**TABLE 3:** Arterial PCO_2_, PO_2_ and mean arterial pressure (MAP) during baseline, hypoxia (HX) and 60 min post-hypoxia for control (CON) and CTB-SAP rats with acute intermittent hypoxia (AIH) or without AIH (time-control or TC). Rats received intrathecal delivery of an inhibitor for tumor necrosis factor alpha receptor 1 (recombinant human soluble TNFR1; sTNFR1i) or vehicle (veh). Significant differences within groups included versus hypoxia (^a^), and 60 min (^b^), and across groups included versus: respective TC (^c^), 28d CON sTNFR1i (^d^), and 28d CTB-SAP sTNFR1i (^e^). Values are expressed as means ± S.E.M. Differences were considered significant if *p* < 0.05.

	P_aCO2_ (mmHg)			P_aO2_ (mmHg)			MAP (mmHg)		
Experimental Groups	baseline	HX	60 min	baseline	HX	60 min	baseline	HX	60 min
With AIH									
Veh									
7d CON	50.1 ± 1.0	49.8 ± 1.2	50.1 ± 1.0	278 ± 13^ab^	37.7 ± 1.8^c^	246 ± 19^a^	126 ± 15^ab^	89 ± 13^c^	120 ± 27^a^
7d CTB-SAP	49.4 ± 0.9	49.3 ± 1.0	48.9 ± 0.7^c^	297 ± 5^abce^	39.7 ± 1.5^c^	268 ± 4^ace^	124 ± 14^ab^	79 ± 12^c^	139 ± 8^ae^
28d CON	51.3 ± 1.1^d^	51.1 ± 1.1^d^	51.1 ± 1.0^d^	286 ± 11^a^	35.7 ± 1.5^c^	270 ± 9^ae^	136 ± 8^a^	99 ± 9	114 ± 10
28d CTB-SAP	51.1 ± 0.8^ad^	49.6 ± 0.8	51.2 ± 0.5^a^	253 ± 25^a^	42.5 ± 3.1^c^	241 ± 23^a^	136 ± 13^ab^	100 ± 10	138 ± 12^ae^
sTNFR1i									
7d CON	48.5 ± 1.8	48.5 ± 1.6	49.0 ± 1.8	283 ± 7^a^	41.7 ± 1.9^c^	267 ± 8^ae^	119 ± 15	95 ± 11	114 ± 16
7d CTB-SAP	51.2 ± 1.2^bd^	51.7 ± 1.1^d^	52.7 ± 1.3^ad^	282 ± 9^ab^	40.1 ± 1.0^c^	257 ± 9^ae^	124 ± 17^ab^	87 ± 12	119 ± 18^ae^
28d CON	47.9 ± 1.8	47.0 ± 1.5	48.4 ± 1.6	285 ± 7^a^	37.5 ± 1.2^c^	265 ± 10^ae^	118 ± 15^ab^	88 ± 13^c^	112 ± 8^a^
28d CTB-SAP	50.5 ± 1.2	50.1 ± 1.6	49.6 ± 1.8	251 ± 24^ab^	38.0 ± 1.1^c^	236 ± 32^a^	115 ± 4^a^	76 ± 9^c^	106 ± 10
Without AIH (TCs)									
CON veh	50.4 ± 1.4	50.2 ± 1.6	50.2 ± 1.3	296 ± 11^b^	296 ± 10	270 ± 9^a^	134 ± 14	137 ± 13	135 ± 13
CTB-SAP veh	50.6 ± 1.8	51.7 ± 1.6	51.5 ± 1.7	233 ± 38	230 ± 45	206 ± 39	111 ± 10	119 ± 13	115 ± 13
CON sTNFR1i	48.8 ± 0.9	48.6 ± 1.1	48.4 ± 0.9	295 ± 9	296 ± 7	278 ± 8	113 ± 14	127 ± 13	114 ± 14
CTB-SAP sTNFR1i	51.9 ± 1.7	51.7 ± 1.6	52.4 ± 2.0	267 ± 20	270 ± 21^b^	245 ± 15	116 ± 11	131 ± 5	123 ± 9

### Short-term hypoxic phrenic responses

**FIGURE 5:**
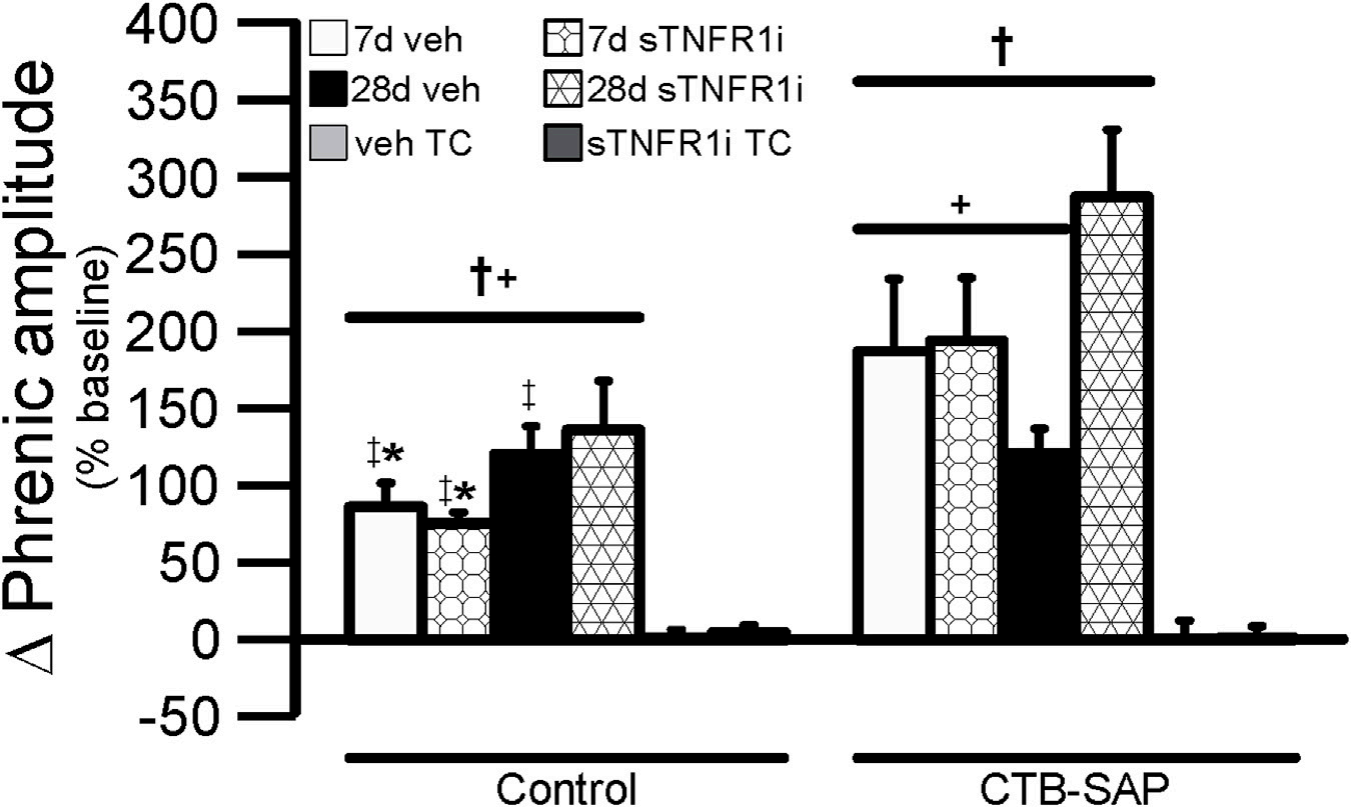
Short-term phrenic nerve hypoxic response (expressed as a percent change from baseline) in 7d (bars with overlaid circles) and 28d (bars with overlaid triangles) control and CTB SAP rats. As predicted, all AIH exposed treatment groups had significantly increased hypoxic response in comparison to the corresponding time control (TC) groups (†). 28d CTB-SAP rats pre-treated with sTNFR1i had an increased phrenic nerve hypoxic response in comparison to all other treatment groups exposed to AIH (+). 7d CTB-SAP rats pre-treated with sTNFR1i had an increased phrenic nerve hypoxic response in comparison to 7d control rats pre-treated with sTNFR1i, 7d control rats pre-treated with vehicle (veh), and 28d control rats pre-treated with veh exposed to AIH (‡). 7d CTB-SAP rats pre-treated with veh had an increased phrenic nerve hypoxic response in comparison to 7d control rats pre-treated with sTNFR1i and 7d control rats pre-treated with veh (*). Values are expressed as means ±1 S.E.M, and differences were considered significant if *p* < 0.05.

Within AIH treated groups phrenic nerve responses were not different across hypoxic episodes (*p* > 0.05; data not shown), so responses during the three bouts of hypoxia were combined in each rat and then compared across treatment groups (Figure 5). Time control (TC) groups not exposed to AIH did not exhibit a significant change in amplitude during the sham AIH exposure as expected (Figure 5; *p* > 0.05). In all AIH-treated groups vs. corresponding TC groups, hypoxic responses were significantly increased (Figure 5; *p* < 0.05). 28d CTB-SAP rats pre-treated with sTNFR1i had an increased phrenic nerve hypoxic response when compared to all other treatment groups exposed to AIH, regardless of time point and pre-treatment, and 7d CTB-SAP rats pre-treated with sTNFR1i had an increased phrenic nerve hypoxic response compared to 7d control rats pre-treated with sTNFR1i, 7d control rats pre-treated with vehicle, and 28d control rats pre-treated with vehicle exposed to AIH (Figure 5; *p* < 0.05). Lastly, 7d CTB-SAP rats pre-treated with vehicle had an increased phrenic nerve hypoxic response compared to 7d control rats pre-treated with sTNFR1i and 7d control rats pre-treated with vehicle (*). Values are expressed as means ±1 S.E.M, and differences were considered significant if *p* < 0.05.

### sTNFR1i reveals enhanced pLTF in 28d CTB-SAP rats, but attenuates pLTF in 7d CTB-SAP rats

**FIGURE 6:**
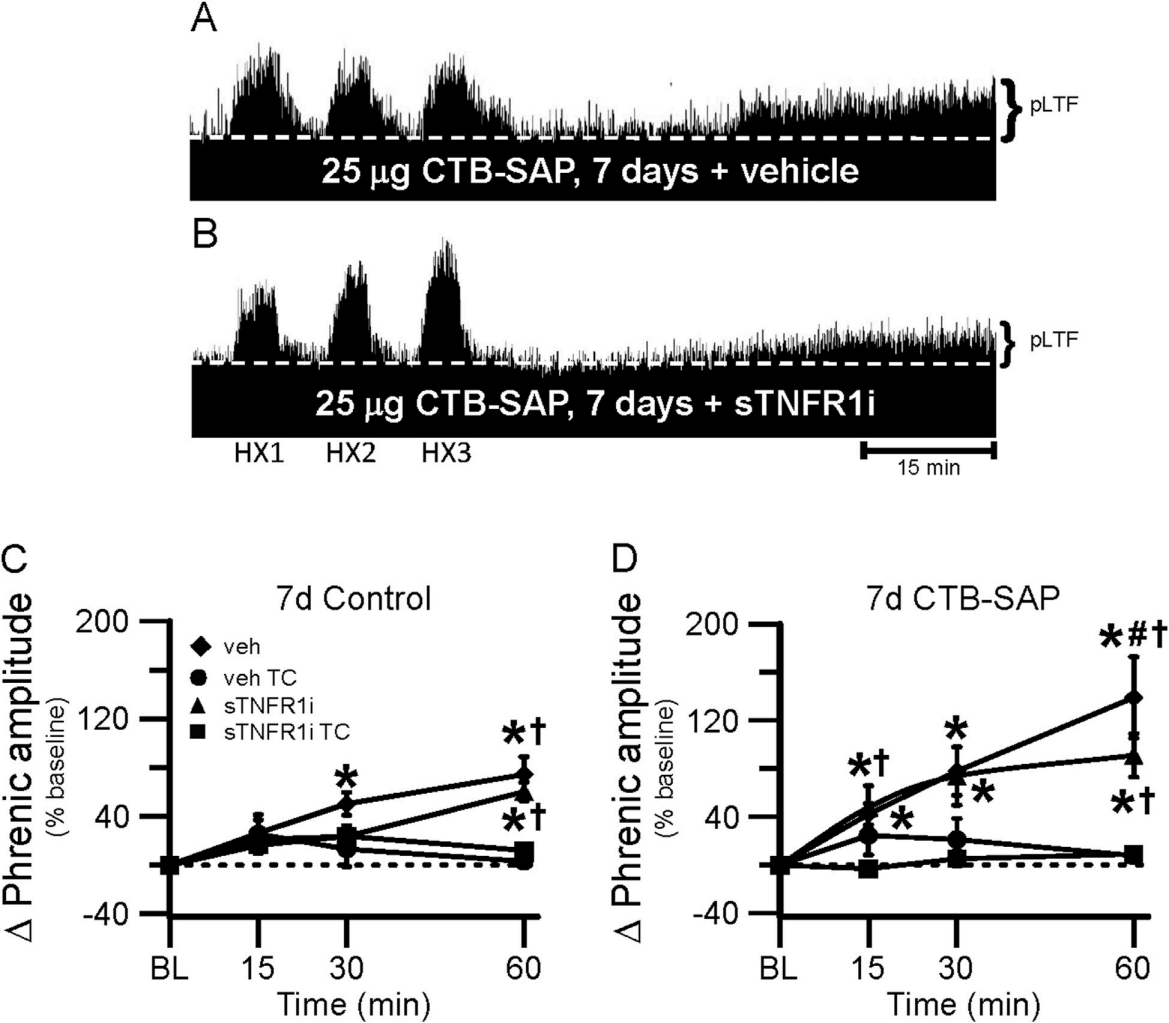
pLTF in 7d rats. A and B, Representative neurograms demonstrating integrated phrenic nerve activity before and after AIH in 7d CTB-SAP rats pre-treated with vehicle (veh; **(A)** or sTNFR1i **(B)**. A white, dashed line indicates baseline on each trace. As expected, 7d CTB-SAP rats pre-treated with veh express enhanced pLTF, while pLTF appears to be attenuated by sTNFR1i pre-treatment. **(C, D)** pLTF (expressed as a percent change from baseline) was significantly increased from baseline (*) at 30 and 60 min post-hypoxia in 7d control rats pre-treated with veh and at 60 min post-hypoxia in those pre-treated with sTNFR1i. pLTF was significantly increased from respective TCs at 15 min (7d CTB-SAP rats pre-treated with veh) and at 60 min in 7d control **(C)** and CTB-SAP **(D)** rats pre-treated with sTNFR1i or veh (†). pLTF was significantly increased from baseline (*) at all time points in 7d CTB-SAP rats pre-treated with veh or with sTNFR1i. 7d CTB-SAP rats pre-treated with veh had a pLTF that was significantly greater than that of 7d CTB-SAP rats pre-treated with sTNFR1i at 60 min post-hypoxia (#). Values are expressed as means ±1 S.E.M, and differences were considered significant if *p* < 0.05.

**FIGURE 7:**
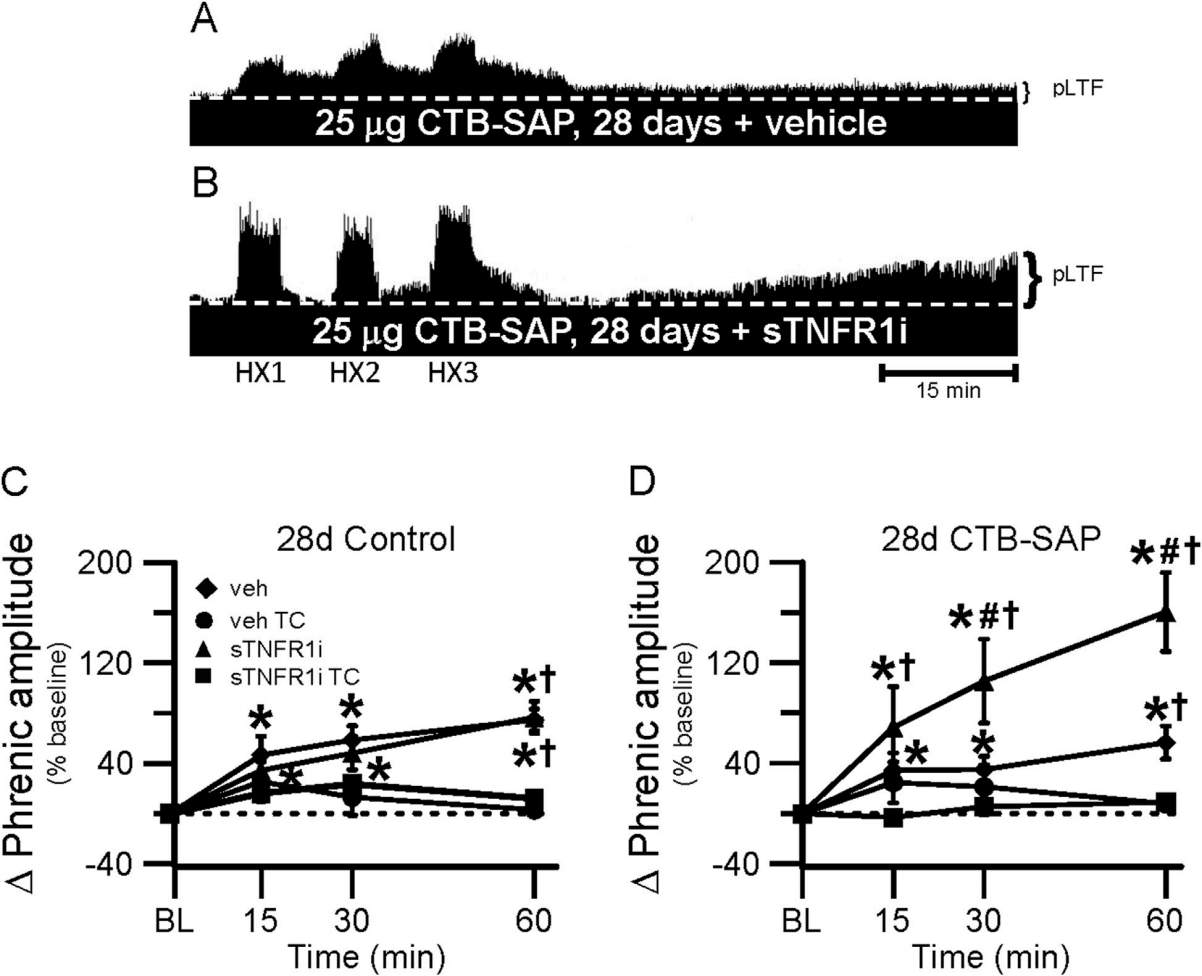
pLTF in 28d rats. A and B, Representative neurograms demonstrating integrated phrenic nerve activity before and after AIH in 28d CTB-SAP rats pre-treated with vehicle (veh; **(A)** or sTNFR1i **(B)**. A white, dashed line indicates baseline on each trace. 28d CTB-SAP rats pre-treated with veh express moderate pLTF as expected, while AIH elicits an enhanced pLTF following sTNFR1i pre-treatment. **(C, D)** pLTF (expressed as a percent change from baseline) was significantly increased from baseline (*) in 28d control and 28d CTB-SAP rats pre-treated with veh and those pre-treated with sTNFR1i at all time points post-hypoxia. pLTF was significantly increased from respective TCs at all time points post-hypoxia in 28d CTB-SAP rats pre-treated with sTNFR1i **(D)**, and at only 60 min post-hypoxia in 28d control **(C)** and CTB-SAP **(D)** rats pre-treated with sTNFR1i or veh (†). pLTF was significantly increased from baseline (*) at all time points in 28d CTB-SAP rats pre-treated with veh or with sTNFR1i. 28d CTB-SAP rats pre-treated with sTNFR1i had a pLTF that was significantly greater than that of 28d CTB-SAP rats pre-treated with veh at 30 and 60 min post-hypoxia (#). Values are expressed as means ±1 S.E.M, and differences were considered significant if *p* < 0.05.

**FIGURE 8:**
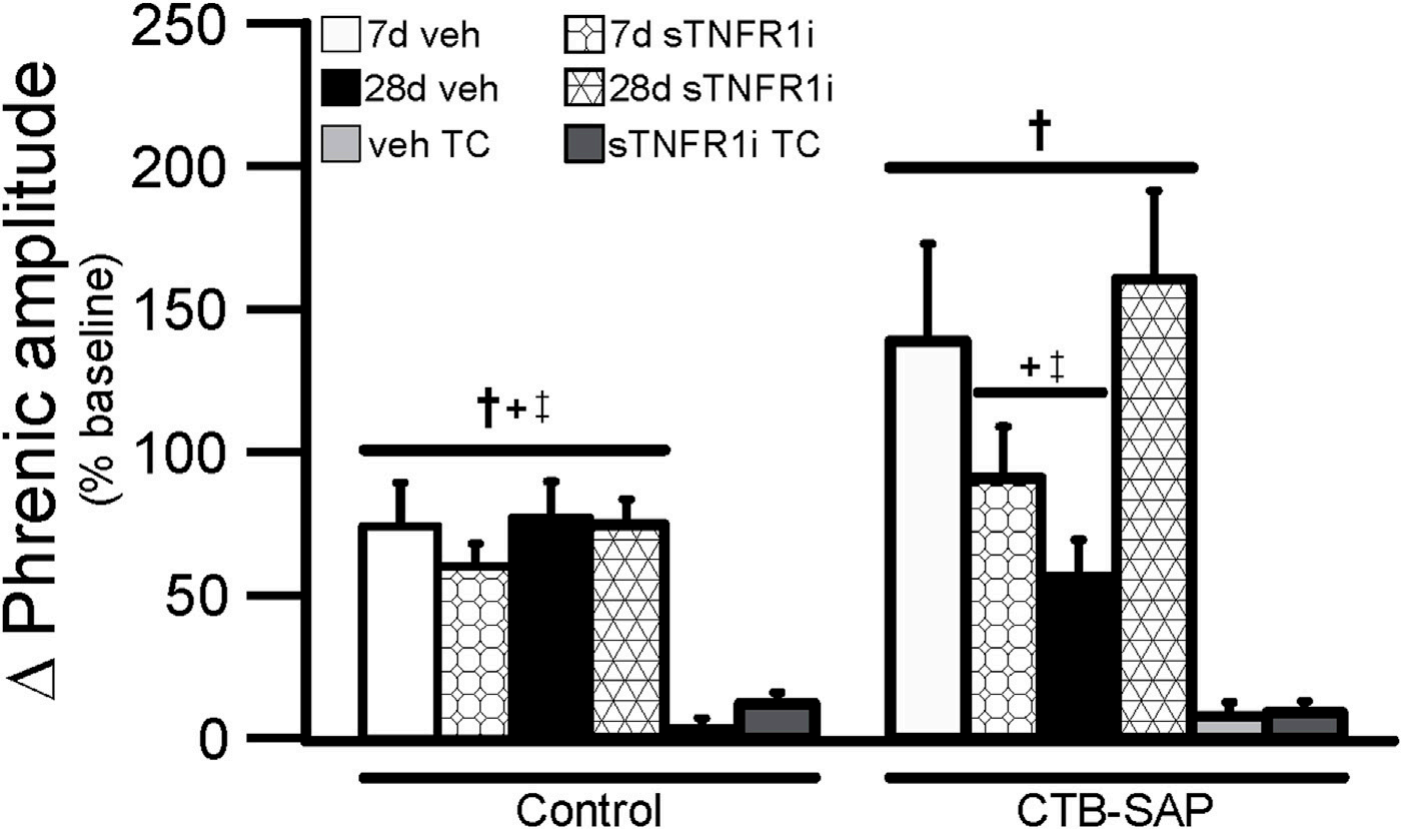
Phrenic amplitude (percent baseline) following AIH at 60 min post-hypoxia in 7d (bars with overlaid circles) and 28d (bars with overlaid triangles) control and CTB-SAP rats. As expected, 7d and 28d control and CTB-SAP rats exhibited greater pLTF compared to the corresponding TC groups (†). pLTF was significantly greater in 7d CTB-SAP rats pre-treated with veh and 28d CTB-SAP rats that were pre-treated with sTNFR1i compared to all control groups, 7d CTB-SAP rats pre-treated with sTNFR1i, and 28d CTB-SAP rats pre-treated with veh (+ and ‡, respectively). Values are expressed as means ± 1 S.E.M, and differences were considered significant if *p* < 0.05.

One goal of this study was to understand if TNF-α signaling plays a role in the differential pLTF observed in CTB-SAP rats. Representative phrenic neurograms are shown for AIH-exposed 7d and 28d CTB-SAP rats and their respective controls approximately 20 min following sTNFR1i or vehicle (aCSF) administration (Figures 6, 7). All groups exposed to AIH elicited pLTF that was significantly increased when compared to baseline and corresponding TCs at 60 min as expected (Figures 6–8; *p* < 0.05). However, pLTF was diminished when 7d CTB-SAP rats were pre-treated with sTNFR1i compared to 7d CTB-SAP rats that had been pre-treated with vehicle (Figures 6, 8; *p* < 0.05), and similar to 7d control rats regardless of pre-treatment (*p* > 0.05). In contrast, 28d CTB-SAP rats pre-treated with sTNFR1i expressed AIH-induced pLTF that was enhanced when compared to 28d CTB-SAP rats pre-treated with vehicle and 28d controls treated with sTNFR1i or vehicle (Figures 7, 8; *p* < 0.05). Interestingly, 28d CTB-SAP rats pre-treated with sTNFR1i expressed enhanced pLTF similar to that of 7d CTB-SAP rats pre-treated with vehicle (Figure 8; *p* > 0.05).

## Discussion

The major findings of this study include: (1) TNFR1 expression is increased on phrenic motor neurons in 28d CTB-SAP rats vs. controls and 7d CTB-SAP rats; (2) astrocytic number is significantly increased in CTB-SAP rats vs. controls, and astrocytes display a reactive (activated) morphology in CTB-SAP rats vs. controls; and (3) TNF-α signaling contributes to the enhanced AIH-induced pLTF observed in 7d CTB-SAP rats, while TNF-α signaling constrains AIH-induced pLTF in 28d CTB-SAP rats. This research provides novel insight into how mechanisms of plasticity are altered over the course of disease progression. Deepening our knowledge of the underlying mechanisms involved and the influence that inflammation has on respiratory plasticity and breathing will allow more precise therapeutic treatments for patients by modulating neuroinflammatory pathways throughout the course of motor neuron loss.

### TNFR1 expression is increased on phrenic motor neurons in 28d CTB-SAP rats

TNF-α is an inflammatory-associated cytokine which acts through two different receptors which often result in opposing biological effects. Under neuroinflammatory conditions, such as multiple sclerosis (MS), tumor necrosis factor receptor-1 (TNFR1) is believed to mediate the pro-inflammatory activities associated with tumor necrosis factor (TNF), while tumor necrosis factor receptor-2 (TNFR2) may induce anti-inflammatory effects and promote remyelination and neuroprotection (Fontaine et al., 2002; Fischer et al., 2020). Here, we looked at TNFR1 expression in the phrenic motor nucleus and the non-phrenic motor nucleus (area immediately surrounding the phrenic motor nucleus) in 7d and 28d CTB-SAP rats and control rats. We found that TNFR1 is significantly increased on phrenic motor neurons in 28d CTB-SAP rats vs. controls and 7d CTB-SAP rats (Figures 1–3; Supplemental Figures S1, 2; *p* < 0.05) but is otherwise unaffected across groups in the phrenic motor nucleus and non-phrenic motor nucleus. Since our previous investigations with this model have shown that inflammation plays beneficial role in respiratory plasticity in 7d CTB-SAP rats, while inflammation constrains respiratory plasticity in 28d CTB-SAP rats (Borkowski et al., 2022), these results suggest a potential therapeutic strategy through TNFR1-selective antagonism in 28d CTB-SAP rats.

### Astrocytic morphological changes are exhibited in the phrenic motor nucleus of CTB-SAP rats

Glial cells (*e.g.,* astrocytes and microglia) are the primary regulators of neuroinflammation. Similar to microglia, astrocytes contribute to maintenance of a wide variety of neurological functions such as synaptic, metabolic, ion and neurotransmitter homeostasis, as well as producing and releasing important inflammatory-associated signaling molecules like TNF-α (Torres-Ceja and Olsen, 2022). Astrocytes undergo phenotypic changes (molecularly and morphologically) in response to injury that are either pro-inflammatory or anti-inflammatory in nature. Both microglia and astrocytes (Table 2) are activated following phrenic motor neuron death inflicted by CTB-SAP injection, suggesting that they could both be contributing to the increased TNF-α release observed in 7d and 28d CTB-SAP rats (Borkowski et al., 2022). Astrocytes are the most abundant cell type in the CNS and under normal conditions, astrocytes are in a resting state, performing multiple functions through ramified branching between synapses. However, upon injury, astrocytes begin to undergo morphological changes characterized by hypertrophy and increased release of pro-inflammatory cytokines. (Wilhelmsson et al., 2006; Chen et al., 2023).

When evaluating the phrenic motor nucleus with IMARIS software, the number of astrocytes was significantly increased in CTB-SAP rats compared to controls (Table 2). Furthermore, an astrocytic morphological analysis conducted using IMARIS software revealed that CTB-SAP rats have significantly increased branch number, number of end points, filament volume, and number of intersections, as well as significantly decreased branch volume and branch length (Figure 4; Table 2; *p* < 0.05) inside the phrenic motor nucleus vs. control rats. Additionally, astrocytic number in the non-phrenic motor nucleus region immediately outside of the phrenic motor nucleus was significantly increased while branch length was significantly decreased (Figure 4; Table 2; *p* < 0.05) in CTB-SAP treated rats vs. controls. Collectively, these findings are indicative of a reactive hypertrophied morphological state in CTB-SAP rats vs. control rats, consistent with observations of astrocyte activation following injury or in states of neurological diseases and models (Wilhelmsson et al., 2006; Hayashi et al., 2022; Chen et al., 2023).

### sTNFR1i delivery constrains pLTF at 7d, while it enhances pLTF in 28d CTB-SAP rats

When bilateral intrapleural injections of CTB-SAP are given, respiratory motor neurons die, but eupnea is maintained (Nichols et al., 2015; Nichols et al., 2018). AIH-induced pLTF has been shown to be enhanced in 7d CTB-SAP rats compared to control rats and 28d CTB-SAP rats (Nichols et al., 2018), and it has been shown that 7d CTB-SAP rats predominately utilize the Gs pathway for pLTF whereas 28d CTB-SAP rats utilize the Gq pathway (Borkowski and Nichols, 2020; Borkowski et al., 2021). However, this enhancement is no longer observed in 7 d in CTB-SAP rats pre-treated with sTNFR1i (Figures 6, 8), while sTNFR1i pre-treatment enhanced pLTF in 28d CTB-SAP rats (Figures 7, 8). Additionally, changes were observed in astrocytic number and morphology at these different time points (Figure 4; Table 2), indicating that they may play a role in the differences in AIH-induced pLTF observed at 7d and 28d.

Our findings suggest that a TNF-α-dependent mechanism plays a role in initiation of enhanced pLTF observed in 7d CTB-SAP rats and is mediated in part by the activation of TNFR1. sTNFR1i did not have an effect on pLTF in control rats. However, when 7d CTB-SAP rats were pre-treated with the TNFR1 inhibitor, sTNFR1i, AIH-induced pLTF was no longer enhanced and appeared similar to that of 7d control rats (Figures 5, 7). Conversely, when 28d CTB-SAP rats were pre-treated with sTNFR1i, AIH-induced pLTF was enhanced to that of 7d CTB-SAP rats that were pre-treated with vehicle (Figures 7, 8). The results from this study show that TNF-α signaling contributes to enhanced pLTF in 7d CTB-SAP rats but constrains pLTF exhibited by 28d CTB-SAP rats. The mechanism(s) by which TNF-α-mediated signaling changes from enhancing pLTF in 7d rats to constraining pLTF in 28d rats following CTB-SAP-induced respiratory motor death remains unknown. However, previous studies have shown that TNF-α signaling in the spinal cord affects spinal plasticity by increasing the trafficking of AMPA receptors to the post-synaptic membrane leading to an upregulation of Ca^2+^ permeable Glutamate receptor 2 (GluR2)-lacking AMPA receptors that ultimately increases postsynaptic excitability (Beattie et al., 2002). TNF-α activity has also been shown to be sufficient for pMF and is required for a form of respiratory plasticity that results from reduction in central respiratory neural activity, known as inactivity-induced phrenic motor facilitation (iPMF) (Baker-Herman and Strey, 2011; Mahamed et al., 2011; Baertsch and Baker-Herman, 2013; Broytman et al., 2013a; Baertsch and Baker-Herman, 2015). Therefore, TNF-α production alone may be able to elicit respiratory plasticity in our model of CTB-SAP-induced respiratory motor neuron loss, which may explain why pLTF enhancement in 7d CTB-SAP rats with TNFR1 inhibition was not observed.

BDNF transcription is upregulated in astrocytes by TNF-α (Saha et al., 2006). We speculate that increased TNF-α expression is contributing to a simultaneous increase in BDNF expression resulting in constrained pLTF levels similar to controls in vehicle treated 28d CTB-SAP rats, which would be consistent with prior studies demonstrating that 28d CTB-SAP rats exhibit BDNF-dependent pLTF (Borkowski and Nichols, 2020). Furthermore, we propose that the reduction of TNF-α and BDNF signaling in 28d CTB-SAP rats pre-treated with sTNFR1i recovers the enhanced pLTF observed in 7d vehicle pre-treated CTB-SAP rats. In addition, suppression of BDNF has been shown to be beneficial when studied in spinal cord injury because BDNF suppression limits astrocyte proliferation and prevents astrogliosis (Tu et al., 2017). Thus, it may be possible that abnormal astrocyte proliferation and cytokine production is constraining pLTF in 28d CTB-SAP rats and is alleviated by inhibition by sTNFR1i of TNF-α dependent BDNF overproduction. This pattern between TNF-α and BDNF expression is not observed in 7d CTB-SAP rats even though TNF-α expression is also significantly increased (Borkowski et al., 2022). We speculate that BDNF overproduction is a result of increased TNF-α signaling following the TNFR1 density increase in 28d CTB-SAP rats causing an increase in reactive astrocyte proliferation and excessive TNF-α release, which have been linked to the exocytosis of non-GluR2 containing AMPARs and an increased vulnerability to excitotoxicity. This vulnerability from increased TNF-α dependent GluR2-lacking AMPA receptor expression is exhibited in ALS models (Stellwagen, 2011) and could be a cause of constrained pLTF in 28d CTB-SAP rats.

## Significance

Despite significant loss of respiratory motor neurons in neuromuscular disorders/diseases such as Pompe disease, progressive muscular atrophy and amyotrophic lateral sclerosis (ALS), breathing capacity is somehow preserved (Bourke et al., 2001; Lyall et al., 2001; Lechtzin et al., 2002; Wong et al., 2002; Nichols et al., 2013; Nichols et al., 2013; Boentert et al., 2017). Although these diseases differ, a common feature is respiratory motor neuron loss, and eventual loss of compensatory mechanisms leading to respiratory muscle paralysis, ventilatory failure and ventilator-dependence or death. There are currently no approved treatment options to significantly and consistently preserve or restore breathing capacity in these patients; thus, there is a critical need of a more effective means to maintain or restore ventilatory function and improve their quality of life. Despite this importance, few studies have investigated how breathing is maintained or therapeutic strategies to preserve or restore ventilation in any neuromuscular disorders or in models of respiratory motor neuron death. Using our established model of bilateral, intrapleural injection of CTB-SAP, we have shown that the TNF-α increase following CTB-SAP-induced phrenic motor loss promotes pLTF in 7d CTB-SAP rats, which can be returned to control levels in the presence of sTNFR1i (Figures 6, 8). However, in 28d CTB-SAP rats when TNFR1 expression is also increased on phrenic motor neurons, pLTF is constrained (Figures 7, 8). This constrained pLTF in 28d CTB-SAP rats can be recovered to the enhanced pLTF level of 7d CTB-SAP rats pre-treated with vehicle when pre-treated with sTNFR1i (Figures 7, 8). Therefore, sTNFR1i may prove to be a potential therapy for 28d CTB-SAP rats and patients in later stages of respiratory motor neuron disease as it may increase the compensatory plasticity of surviving phrenic motor neurons which may improve motor neuron function, breathing, and quality of life. Future studies will focus on the effect cytokines released from microglia and astrocytes have on the differential mechanisms of pLTF utilized by 7d and 28d CTB-SAP rats and potential therapies that target them such as the pharmacological effects of chronic sTNFR1i administration.

## Data Availability

The raw data supporting the conclusions of this article will be made available by the authors, without undue reservation.
