# Corrigendum: Spinal TNF-α receptor 1 is differentially required for phrenic long-term facilitation (pLTF) over the course of motor neuron death in adult rats

**DOI:** 10.3389/fphys.2025.1597278

**Published:** 2025-04-01

**Authors:** Ryan D. Lewis, Amy N. Keilholz, Catherine L. Smith, Ethan A. Burd, Nicole L. Nichols

**Affiliations:** ^1^ Department of Biological Chemistry, Grinnell College, Grinnell, IA, United States; ^2^ Department of Veterinary Pathobiology, College of Veterinary Medicine, University of Missouri, Columbia, MO, United States; ^3^ Department of Biomedical Sciences, College of Veterinary Medicine, University of Missouri, Columbia, MO, United States; ^4^ Department of Biology, Seton Hill University, Greensburg, PA, United States; ^5^ Department of Medical Pharmacology and Physiology, School of Medicine, University of Missouri, Columbia, MO, United States; ^6^ Dalton Cardiovascular Research Center, University of Missouri, Columbia, MO, United States

**Keywords:** phrenic motor neuron death, breathing, respiration, plasticity, astrocyte, rat model

In the published article, there was an error in the **Funding** statement. The funding statement regarding the Computational Neuroscience grant from the National Science Foundation was displayed as “RDL and EAB were supported by a Computational Neuroscience grant from the National Science Foundation”. The correct statement is “RDL and EAB were supported by a Computational Neuroscience grant from the National Science Foundation (NSF DBI 1950787),”]. The correct **Funding** statement appears below.

