# Supplementary figures and images for: Spinal TNF-α receptor 1 is differentially required for phrenic long-term facilitation (pLTF) over the course of motor neuron death in adult rats
Source: Front Physiol. 2024 Dec 5;15:1488951. doi: 10.3389/fphys.2024.1488951 (PMC11656052; doi:10.3389/fphys.2024.1488951)

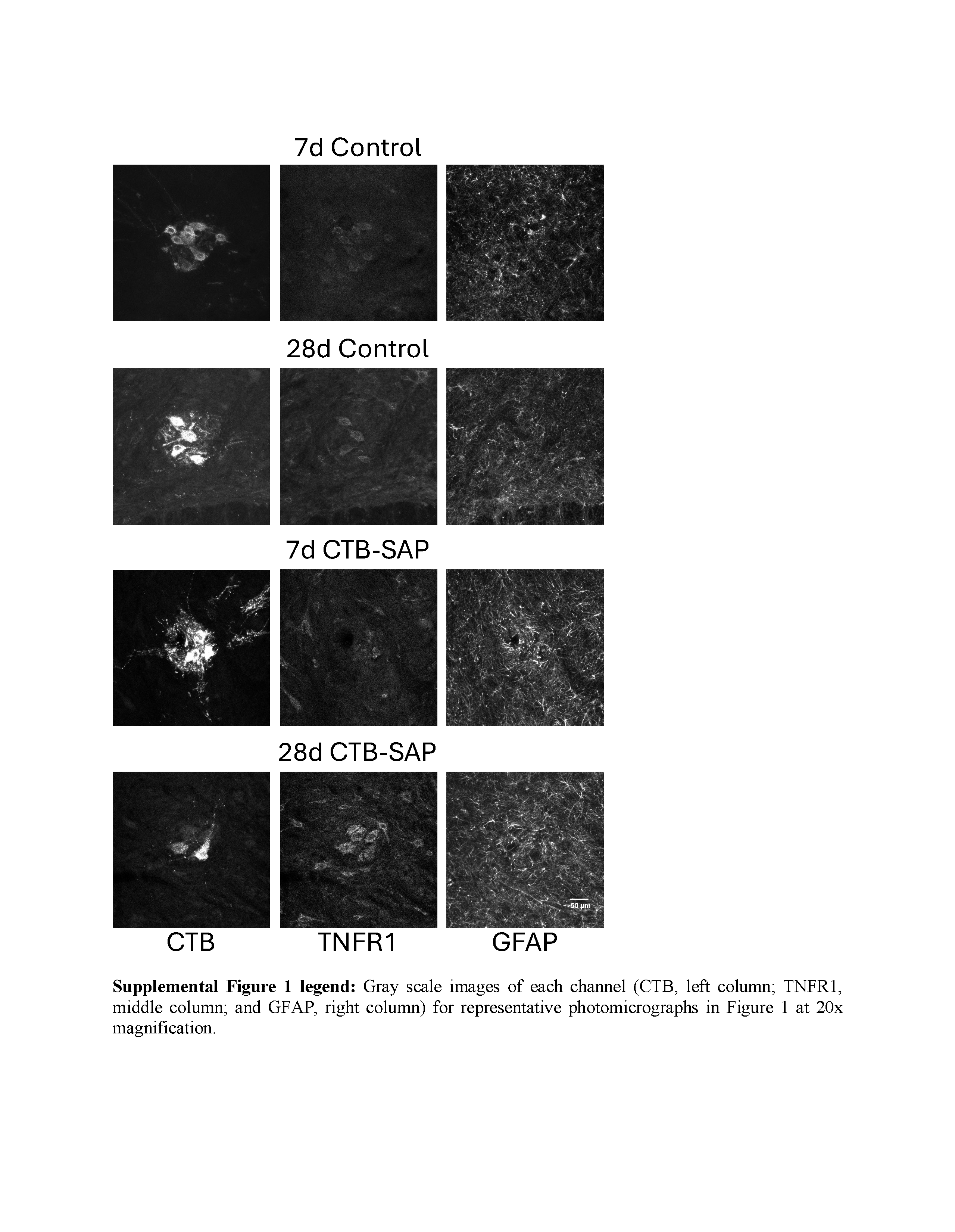

Supplement: Supplementary file 1 [file Image1.tiff]

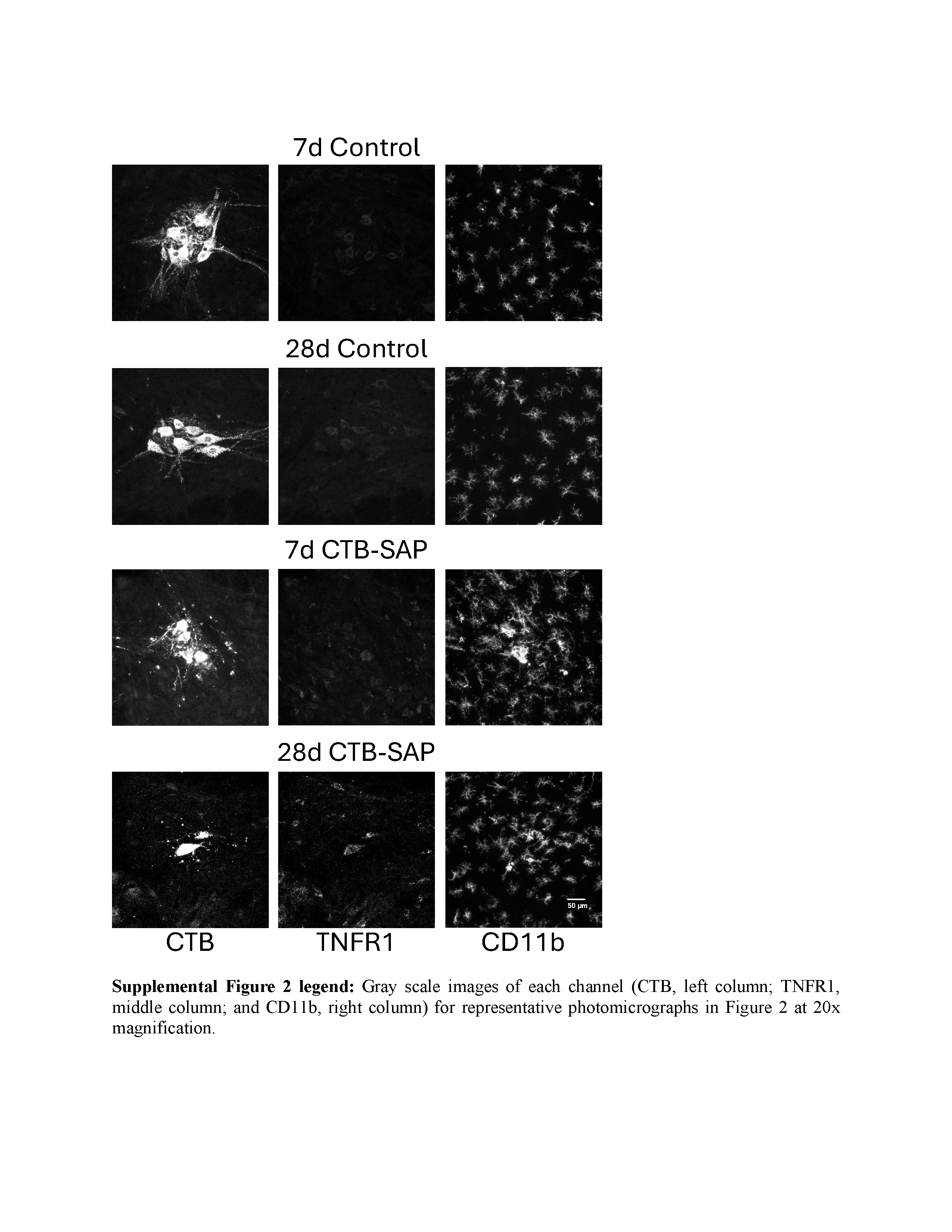

Supplement: Supplementary file 2 [file Image2.tiff]
